# The genome sequence of the Common Wave, *Cabera exanthemata* (Scopoli, 1763) (Lepidoptera: Geometridae)

**DOI:** 10.12688/wellcomeopenres.27917.1

**Published:** 2026-09-24

**Authors:** Charlotte J. Wright, Witold Morek, Darwin Tree of Life Barcoding Collective, Wellcome Sanger Institute Tree of Life Management, Samples and Laboratory team, Wellcome Sanger Institute Scientific Operations: Sequencing Operations, Wellcome Sanger Institute Tree of Life Core Informatics team, Tree of Life Core Informatics collective, Darwin Tree of Life Consortium

**Affiliations:** 1 Wellcome Sanger Institute, Hinxton, Cambridgeshire, England, UK

**Keywords:** Cabera exanthemata, Common Wave, genome sequence, chromosomal, Lepidoptera

## Abstract

We present a genome assembly from an individual male *Cabera exanthemata* (Common Wave; Arthropoda; Insecta; Lepidoptera; Geometridae). The assembly contains two haplotypes with total lengths of 525.76 megabases and 522.27 megabases. Most of the haplotype 1 assembly (99.6%) was scaffolded into 31 chromosomal pseudomolecules, including the Z sex chromosome. Haplotype 2 was assembled to scaffold level. The mitochondrial genome has also been assembled, with a length of 15.43 kilobases. This assembly was generated as part of the Darwin Tree of Life project, which produces genomes for eukaryotic species found in Britain and Ireland.

## Species taxonomy

Eukaryota; Opisthokonta; Metazoa; Eumetazoa; Bilateria; Protostomia; Ecdysozoa; Panarthropoda; Arthropoda; Mandibulata; Pancrustacea; Altocrustacea; Allotriocarida; Hexapoda; Insecta; Dicondylia; Pterygota; Neoptera; Eumetabola; Endopterygota; Aparaglossata; Panorpida; Amphiesmenoptera; Lepidoptera; Glossata; Neolepidoptera; Heteroneura; Ditrysia; Obtectomera; Geometroidea; Geometridae; Ennominae; *Cabera*; *Cabera exanthemata* (Scopoli, 1763) (NCBI:txid934826)

## Background

*Cabera exanthemata* (Scopoli, 1763), the Common Wave, is a geometrid moth with a Holarctic distribution. It is generally common in Central Europe and is widespread and common throughout Britain and Ireland ( Wagner, 2026; Waring & Townsend, 2017).

Adults have a forewing length of 14–16 mm. The species resembles the Common White Wave, but its cross-lines are brown, often indistinct and more strongly curved, particularly the outer two (Figure 1). Brownish-grey freckling varies in intensity and is usually heavier in females. Individuals of the second generation are often smaller and darker, with reduced cross-banding, and the species can also be confused with the grass wave ( Waring & Townsend, 2017).

In Britain, *C. exanthemata* has two generations at least as far north as Lancashire, flying from May to early July and again from early July to early September. In Scotland, the moth has been recorded continuously from May to September, peaking between mid-June and early July, and later records probably represent a partial second generation. Adults rest among the foliage of their larval foodplants during the day and are easily disturbed. They fly mainly at dusk, visit flowers such as wild privet and are attracted to light, although they are generally more numerous at dusk. The larvae feed on sallows, including goat willow and grey willow, as well as aspen and other poplars. The species inhabits woodland and carr, particularly wet sites, and overwinters as a pupa among plant debris or near the soil surface ( Waring & Townsend, 2017).

We present a chromosome-level genome sequence for *Cabera exanthemata*, produced using the Tree of Life pipeline. The sequence is based on a specimen collected from Hinxton, Cambridgeshire, England, UK. This assembly was generated as part of the Darwin Tree of Life Project, which aims to generate high-quality reference genomes for all named eukaryotic species in Britain and Ireland to support research, conservation, and the sustainable use of biodiversity ( Darwin Tree of Life Project Consortium, 2022).

**Figure 1.**
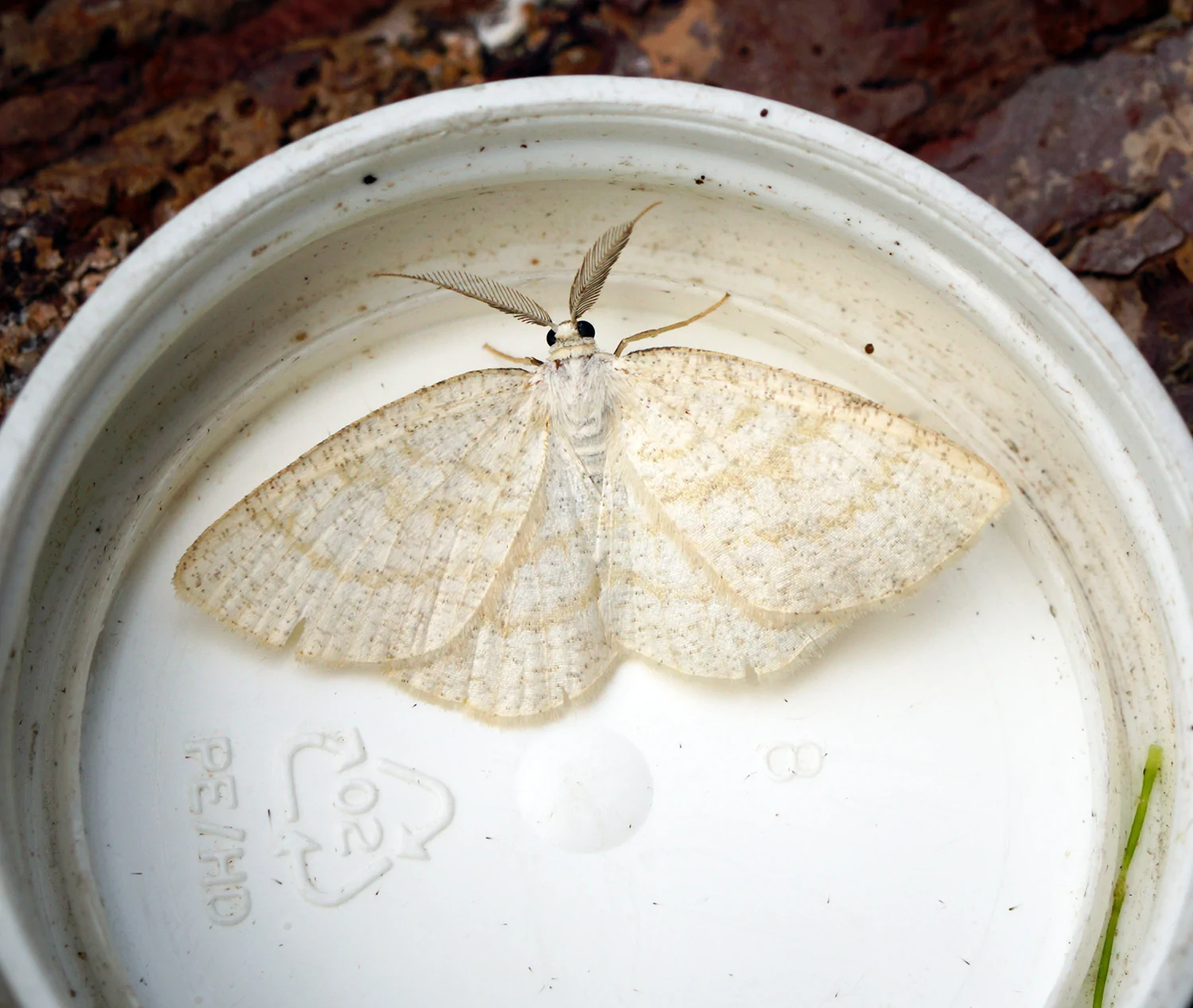
** *Cabera exanthemata* photographed by Ben Sale; not the specimen used for sequencing.

## Methods

### Sample acquisition

The specimen used for genome sequencing was an adult male *Cabera exanthemata* (specimen ID SAN00003356, ToLID ilCabExan1). The same specimen was also used for Hi-C sequencing. It was collected from Hinxton, Cambridgeshire, England, UK (latitude 52.0749, longitude 0.1843) on 2023-08-08 by Charlotte Wright and Witold Morek and identified by Charlotte Wright.

### Nucleic acid extraction

High molecular weight (HMW) DNA extraction protocols developed by the Tree of Life Core Laboratory at the Wellcome Sanger Institute (WSI) are available on protocols.io ( Howard *et al.*, 2025). Tissue from the ilCabExan1 sample was weighed and triaged to select the appropriate extraction workflow. For library DTOL15016444, 11.0 mg of thorax tissue was used. The tissue was homogenised following PowerMash. HMW DNA was extracted using the Automated MagAttract v2 protocol. DNA was sheared into an average fragment size of 12–20 kb following the Megaruptor®3 for LI PacBio protocol. Sheared DNA was purified by automated SPRI (solid-phase reversible immobilisation).

The concentration of the sheared and purified DNA was assessed with a NanoDrop spectrophotometer and a Qubit Fluorometer using the Qubit dsDNA High Sensitivity Assay kit. Fragment size distribution was evaluated by running the sample on the Femto Pulse system. For this sample, final post-shearing DNA measurements included a Qubit concentration of 29.56 ng/μL, a yield of 1,389.32 ng, and an average fragment size of 15.9 kb. The 260/280 spectrophotometric ratio was 1.97, and the 260/230 ratio was 2.47. The Genomic Quality Number (GQN) was 7.4.

### PacBio HiFi library preparation and sequencing

Library preparation and sequencing took place in the WSI Scientific Operations core. Library DTOL15016444 was prepared with the SMRTbell Prep Kit 3.0 (Pacific Biosciences) according to the manufacturer’s instructions. The kit includes reagents for end repair/A-tailing, adapter ligation, post-ligation SMRTbell bead clean-up, and nuclease treatment. Size selection and clean-up were performed using diluted AMPure PB beads (Pacific Biosciences). DNA concentration was quantified with a Qubit Fluorometer v4.0 (Thermo Fisher Scientific) and the Qubit 1X dsDNA HS assay kit. Final library fragment size was assessed with the Agilent Femto Pulse Automated Pulsed Field CE Instrument (Agilent Technologies) using the gDNA 55 kb BAC analysis kit.

The library was sequenced on a Revio instrument (Pacific Biosciences, California, USA). The prepared libraries selected for multiplexing were pooled based on genome size, library molarity and the intended plex level. Primers were annealed and polymerases were bound to generate circularised complexes following the manufacturer’s instructions. Complexes were purified using SMRTbell beads, diluted to the Revio loading concentration, and spiked with a Revio sequencing internal control. The pooled libraries were sequenced on a Revio 25M Plex SMRT cell in a 2-plex run. SMRT Link software (Pacific Biosciences) was used to configure and monitor the run and to perform primary and secondary data analysis.

### Hi-C

***Sample preparation and crosslinking***

Hi-C data were generated from the ilCabExan1 head sample using the Arima-HiC v2 kit (Arima Genomics). Following the manufacturer’s instructions, tissue was fixed and DNA crosslinked using TC buffer to a final formaldehyde concentration of 2%. The tissue was homogenised using the Diagnocine Power Masher-II. Crosslinked DNA was digested with a restriction enzyme master mix, biotinylated, and ligated. Clean-up was performed with SPRISelect beads before library preparation. DNA concentration was measured with the Qubit Fluorometer (Thermo Fisher Scientific) and Qubit HS Assay Kit. The biotinylation percentage was estimated using the Arima-HiC v2 QC beads.

***Hi-C library preparation and sequencing***

Biotinylated DNA constructs were fragmented using a Covaris E220 sonicator and size-selected to 400–600 bp using SPRISelect beads. DNA was enriched with Arima-HiC v2 kit Enrichment beads. End repair, A-tailing, and adapter ligation were carried out with the NEBNext Ultra II DNA Library Prep Kit (New England Biolabs), following a modified protocol where library preparation occurs while DNA remains bound to the Enrichment beads. Libraries were amplified with KAPA HiFi HotStart mix and a custom Unique Dual Index (UDI) barcode set (Integrated DNA Technologies). Depending on sample concentration and biotinylation percentage determined at the crosslinking stage, libraries were amplified with 10–16 PCR cycles. Post-PCR clean-up was performed with SPRISelect beads. Libraries were quantified using the AccuClear Ultra High Sensitivity dsDNA Standards Assay Kit (Biotium) and a FLUOstar Omega plate reader (BMG Labtech).

Prior to sequencing, libraries were normalised to 10 ng/μL. Normalised libraries were quantified again to create equimolar and/or weighted 2.8 nM pools. Pool concentrations were checked using the Agilent 4200 TapeStation (Agilent) with High Sensitivity D500 reagents before sequencing. Libraries were sequenced on the Illumina NovaSeq X, generating paired-end 150-bp reads.

### Genome assembly

Prior to assembly of the PacBio HiFi reads, a database of *k*-mer counts ( *k* = 31) was generated from the filtered reads using FastK. The *k*-mer frequency distribution was analysed with GenomeScope (version 2.1.0) ( Ranallo-Benavidez *et al.*, 2020), providing fitted point estimates of haploid genome size, heterozygosity and repeat content.

The HiFi reads were assembled using Hifiasm in Hi-C phasing mode ( Cheng *et al.*, 2021, 2022), producing two haplotypes. Hi-C reads ( Rao *et al.* , 2014) were mapped to contigs using bwa-mem2 ( Vasimuddin *et al.*, 2019). Contigs were further scaffolded with Hi-C data in YaHS ( Zhou *et al.*, 2023), using the --break option for handling potential misassemblies. The scaffolded assemblies were evaluated using Gfastats ( Formenti *et al.* , 2022), BUSCO ( Manni *et al.*, 2021) and MerquryFK ( Rhie *et al.*, 2020).

The mitochondrial genome was assembled using MitoHiFi ( Uliano-Silva *et al.* , 2023). The MitoHiFi reference was the mitochondrial genome of *Biston thoracicaria* (NC_057638.1).

### Assembly curation

The assembly was decontaminated using the Assembly Screen for Cobionts and Contaminants ( ASCC) pipeline. The flat files and maps for curation were generated with TreeVal. Manual curation was conducted primarily in PretextView and HiGlass ( Kerpedjiev *et al.* , 2018). Scaffolds were visually inspected and corrected as described by Howe *et al*. (2021). The two haplotype assemblies were combined for curation. Manual corrections included ten breaks and 59 joins. This reduced the scaffold count by 24.4%. The curation process is described at sanger-tol/curation-resources. A Hi-C contact map of the haplotype 1 assembly was generated with PretextSnapshot.

### Assembly quality assessment

The MerquryFK tool ( Rhie *et al.*, 2020) was run in a Singularity container ( Kurtzer *et al.*, 2017) to evaluate *k*-mer completeness and assembly quality for both haplotypes using the *k*-mer database ( *k* = 31) computed prior to genome assembly. The analysis outputs included assembly QV scores and completeness statistics.

The haplotype 1 assembly was assessed using BlobToolKit ( Challis *et al.*, 2020). PacBio reads were mapped to the assembly to derive sequence coverage, and BUSCO ( Manni *et al.*, 2021) was used to assess gene-set completeness. DIAMOND BLASTp searches of extracted BUSCO proteins and DIAMOND BLASTx searches of assembly sequences against UniProt Reference Proteomes ( Buchfink *et al.*, 2021), followed by BLASTn searches of sequences without DIAMOND BLASTx hits against the NCBI nt database ( Altschul *et al.*, 1990), supported taxonomic assignment. Coverage, sequence composition, taxonomic assignment and BUSCO results were integrated into a BlobDir for interactive inspection and visualisation. The snail and blob plots were rendered locally from this hosted BlobDir using BlobTk v0.8.3. The scaffold-length radial scale function for the snail plot was set to linear ( Challis & Blaxter, 2026). The blob plot used circular markers with area proportional to scaffold length ( Challis *et al.*, 2020).

Ancestral linkage groups were painted along the chromosome-level scaffolds of the haplotype 1 assembly. Complete BUSCO genes (single-copy and duplicated) from the lepidoptera_odb10 lineage dataset were assigned to the 32 Lepidoptera Merian elements (MZ and M1–M31) described by Wright *et al.*. (2024) and plotted along chromosomes drawn to scale.

## Genome sequence report

### Sequence data

PacBio sequencing of the *C. exanthemata* specimen generated 41.27 Gb (gigabases) from 3.98 million reads. These reads were used to assemble the genome. GenomeScope analysis using a *p* = 2 model gave a *k*-mer-based haploid genome size estimate of 519.78 Mb, with heterozygosity of 1.27% and repeat content of 27.24%; the full-model fit was 97.46% (Figure 2). The GenomeScope estimates guided expectations for the assembly. The NCBI assembly record reports 76× genome coverage. Hi-C sequencing produced 130.53 Gb from 432.22 million reads, which were used to scaffold the haplotype 1 assembly. Table 1 summarises the specimen and sequencing details.

**Figure 2.**
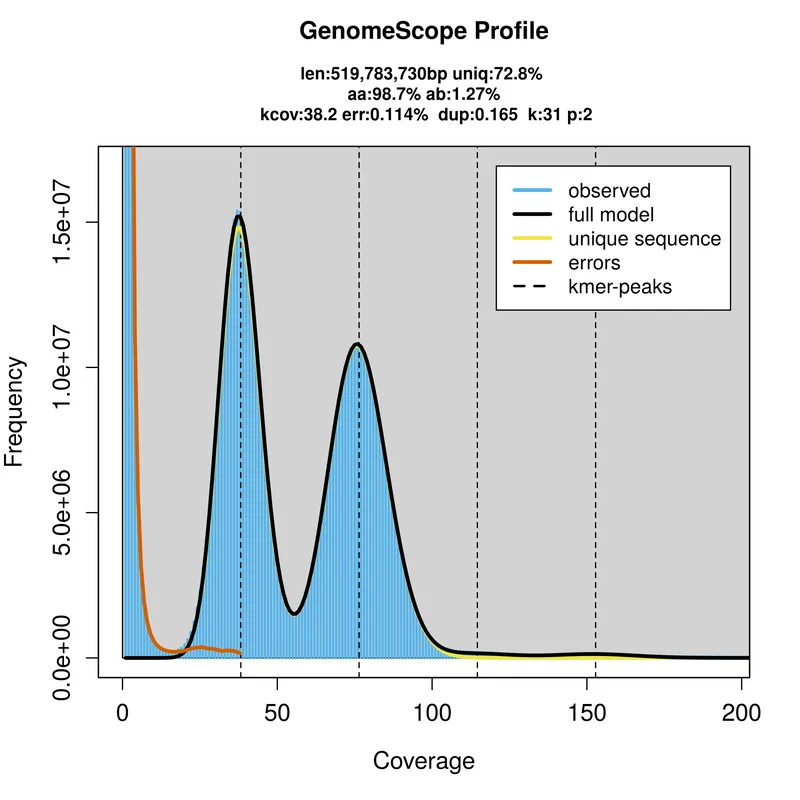
GenomeScope model fitted to the *k*-mer frequency distribution. The plot shows the observed and modelled *k*-mer spectra used to estimate haploid genome size, heterozygosity and repeat content from unassembled sequencing reads.

**Table 1.** Specimen and sequencing data for *C. exanthemata* (BioProject PRJEB82149)

**Platform**	**PacBio HiFi**	**Hi-C**
**ToLID**	ilCabExan1	ilCabExan1
**Specimen ID**	SAN00003356	SAN00003356
**BioSample (source individual)**	SAMEA115163566	SAMEA115163566
**BioSample (tissue)**	SAMEA115163599	SAMEA115163595
**Tissue**	thorax	head
**Instrument**	Revio	Illumina NovaSeq X
**Library ID & run accession(s)**	DTOL15016444: ERR13946452	ERR13947471
**Read count total**	3.98 million reads	432.22 million read pairs
**Base count total**	41.27 Gb	130.53 Gb

### Assembly statistics

The genome was assembled into two haplotypes using Hi-C phasing. The haplotype 1 assembly was curated to chromosome level, while haplotype 2 was assembled to scaffold level. The haplotype 1 assembly, which is proposed as the reference assembly for this species, has a total length of 525.76 Mb in 95 scaffolds (excluding organelle sequences), with 83 gaps, and a scaffold N50 of 18.52 Mb (Table 2). The haplotype 1 assembly has a scaffold auN of 17.6 Mb.

**Table 2.** Genome assembly statistics for *C. exanthemata*

**Genome assembly**	**Haplotype 1**	**Haplotype 2**
**Assembly name**	ilCabExan1.hap1.1	ilCabExan1.hap2.1
**Assembly accession**	GCA_964340555.1	GCA_964340675.1
**Assembly level**	chromosome	scaffold
**Span (Mb)**	525.76	522.27
**Number of chromosomes**	31	-
**Number of contigs**	178	136
**Contig N50 (Mb)**	10.27	9.34
**Number of scaffolds (excluding organelle sequences)**	95	52
**Scaffold N50 (Mb)**	18.52	18.13
**Scaffold auN (Mb)**	17.6	17.6
**Longest scaffold length (Mb)**	23.3	23.71
**Sex chromosomes**	Z	-
**Organelles**	Mitochondrial genome: 15.43 kb	-

Most of the haplotype 1 assembly sequence (99.6%) was assigned to 31 chromosome-level scaffolds, representing 30 autosomes and the Z sex chromosome. These chromosome-level scaffolds, confirmed by Hi-C data, are named according to size (Figure 3; Table 3). Chromosome Z was assigned by mapping of Merian elements.

The mitochondrial genome was also assembled (length 15.43 kb, OZ203801.1). This sequence is included as a contig in the multifasta file of the haplotype 1 genome submission and as a standalone record.

**Figure 3.**
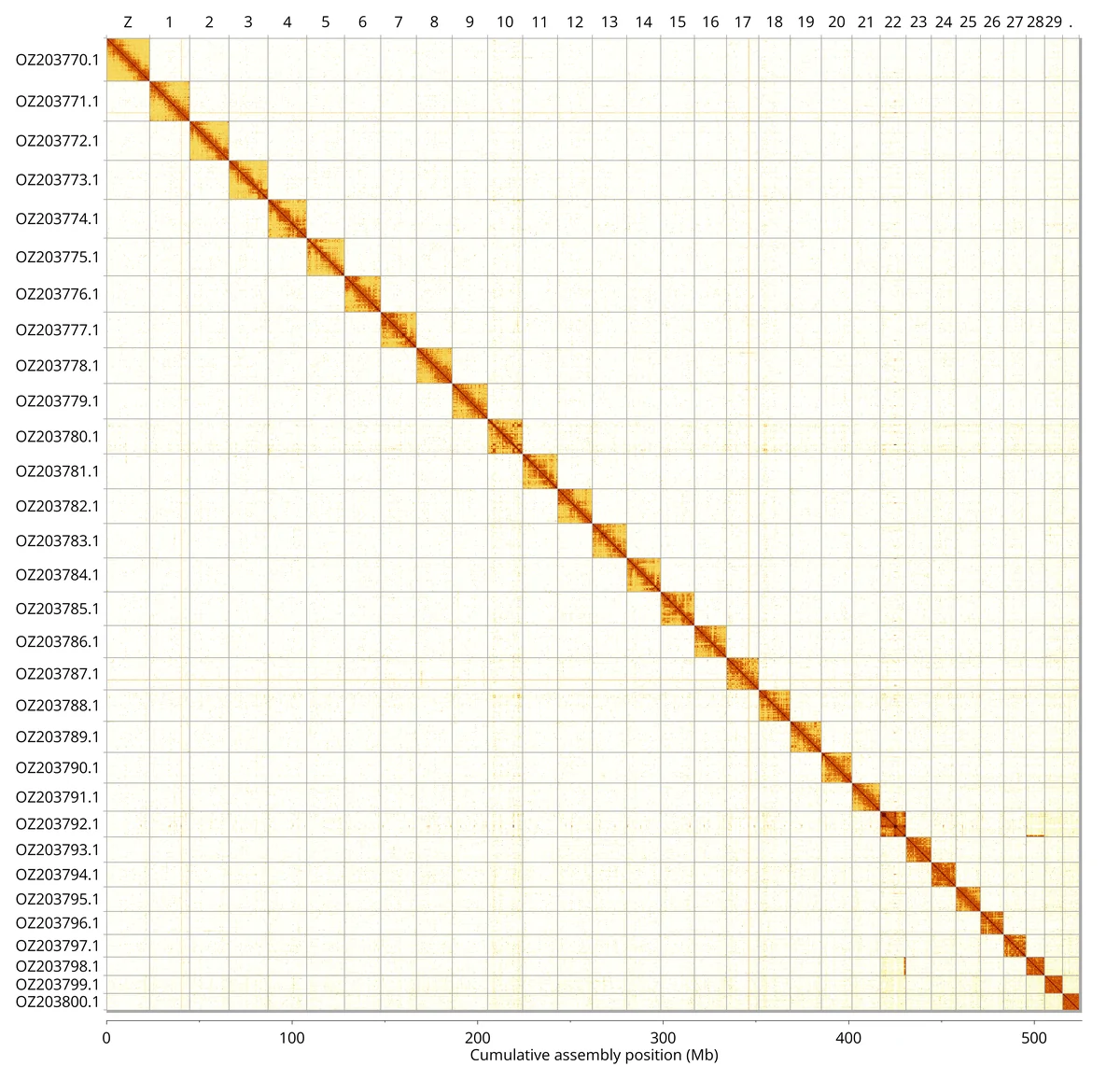
Hi-C contact map of the *C. exanthemata* haplotype 1 assembly. Assembled chromosomes are shown in order of size and labelled along the axes, with a megabase scale shown below. The plot was generated using PretextSnapshot.

**Table 3.** Chromosomal pseudomolecules in the haplotype 1 genome assembly of *C. exanthemata* ilCabExan1

**INSDC accession**	**Molecule**	**Length (Mb)**	**GC%**
OZ203771.1	1	21.52	36
OZ203772.1	2	21.13	36
OZ203773.1	3	20.94	36
OZ203774.1	4	20.92	35.50
OZ203775.1	5	20.34	36
OZ203776.1	6	19.44	36
OZ203777.1	7	19.31	35.50
OZ203778.1	8	19.17	36
OZ203779.1	9	19.12	36
OZ203780.1	10	18.86	36.50
OZ203781.1	11	18.86	35.50
OZ203782.1	12	18.69	36
OZ203783.1	13	18.52	36
OZ203784.1	14	18.20	36
OZ203785.1	15	18.17	36
OZ203786.1	16	17.38	36
OZ203787.1	17	17.32	36
OZ203788.1	18	16.86	36
OZ203789.1	19	16.76	36.50
OZ203790.1	20	16.53	36
OZ203791.1	21	15.15	36
OZ203792.1	22	13.90	38.50
OZ203793.1	23	13.65	36
OZ203794.1	24	13.27	36
OZ203795.1	25	13.23	36.50
OZ203796.1	26	12.42	36
OZ203797.1	27	12.16	36
OZ203798.1	28	9.84	36
OZ203799.1	29	9.68	36.50
OZ203800.1	30	9.02	36
OZ203770.1	Z	23.30	36

Chromosome painting shows the distribution of Lepidoptera Merian elements assignments across the chromosome-level scaffolds of the haplotype 1 assembly (Figure 4).

**Figure 4.**
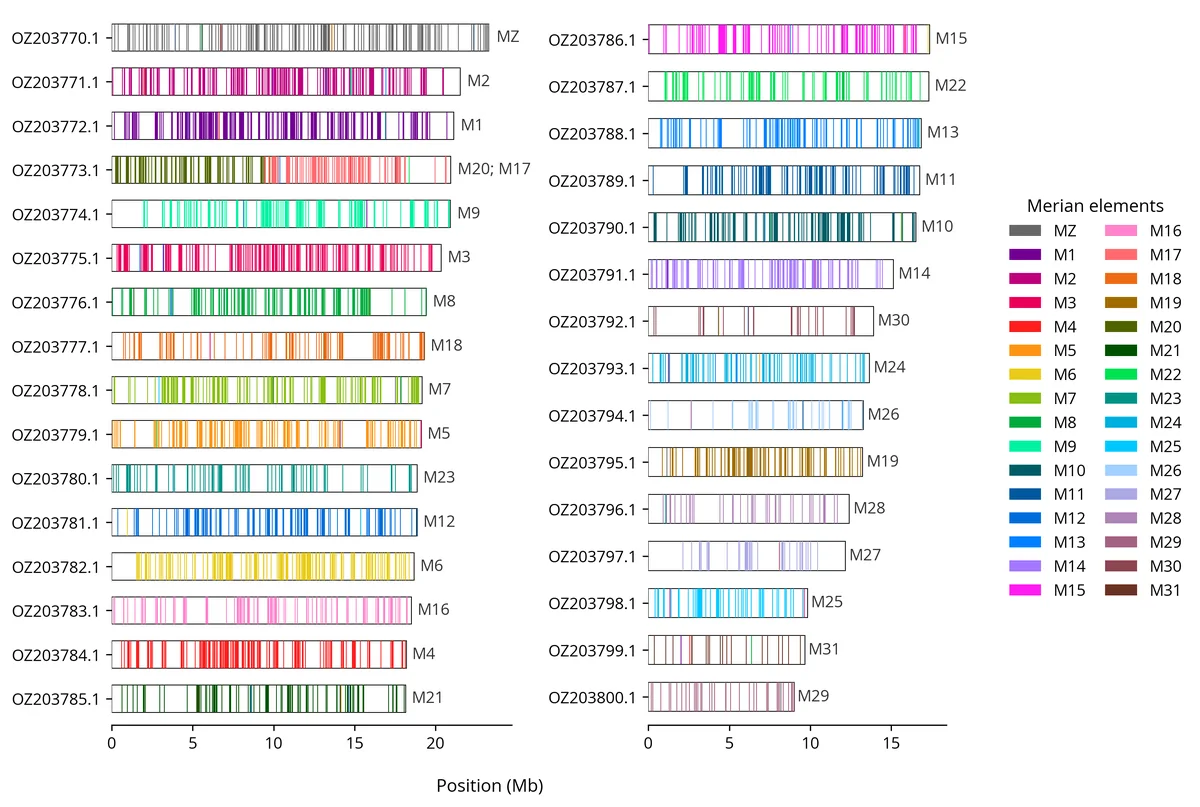
Lepidoptera Merian elements painting of the haplotype 1 assembly of *C. exanthemata*. Chromosomes are drawn to scale, with coloured bars marking the positions of complete lepidoptera_odb10 BUSCO genes. Each BUSCO gene is coloured by its assignment to one of the 32 Merian elements (MZ and M1–M31).

### Assembly quality metrics

For the haplotype 1 assembly, the estimated QV is 65.5, and for the haplotype 2 assembly, 67.6. When the two haplotypes are combined, the assembly achieves an estimated QV of 66.4. The *k*-mer completeness is 75.57% for the haplotype 1 assembly, 75.52% for the haplotype 2 assembly, and 99.70% for the combined haplotypes (Figure 5).

**Figure 5.**
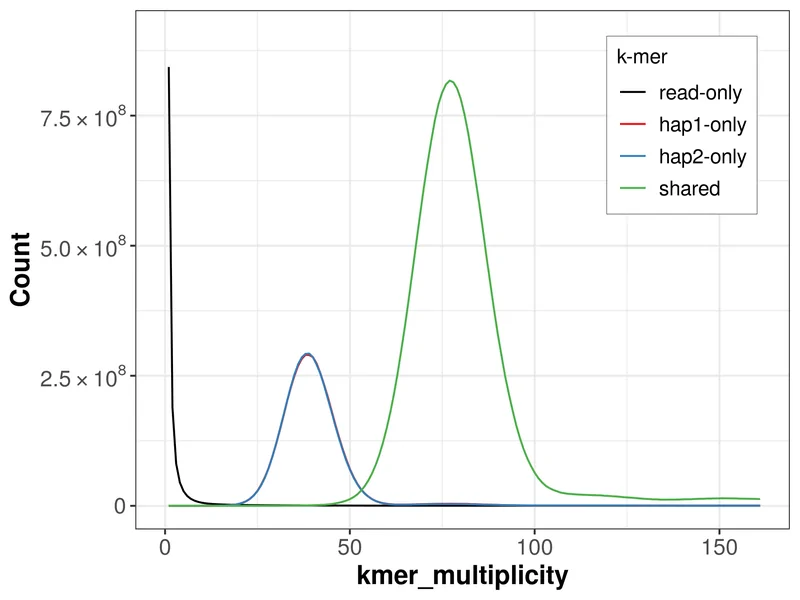
Evaluation of *k*-mer completeness using MerquryFK. This plot illustrates the recovery of *k*‐mers from the original read data in the haplotype 1 assembly, the haplotype 2 assembly and the combined haplotypes. The horizontal axis represents *k*‐mer multiplicity, and the vertical axis shows the number of *k*‐mers. The black curve represents *k*‐mers that appear in the reads but are not assembled. The green curve corresponds to *k*‐mers shared by both haplotypes, and the red and blue curves show *k*‐mers found only in one of the haplotypes.

BUSCO analysis using the lepidoptera_odb10 reference set ( *n* = 5,286) identified 98.4% of the expected gene set (single = 97.9%, duplicated = 0.5%) in the haplotype 1 assembly. For the haplotype 2 assembly, BUSCO v.5.5.0 analysis identified 98.4% of the expected gene set (single = 97.9%, duplicated = 0.5%).

The snail plot in Figure 6 provides a summary of assembly contiguity, cumulative scaffold count, sequence composition and BUSCO completeness for the haplotype 1 assembly ( Challis & Blaxter, 2026). The relative sizes of the longest scaffold, scaffold N50 and N90, together with the dark-grey scaffold-length distribution, show how much of the assembly is contained in long scaffolds. The outer composition track shows variation in GC, AT and N content, while the central track shows the cumulative scaffold count and the BUSCO inset summarises gene-set completeness.

The blob plot in Figure 7 displays scaffolds from the haplotype 1 assembly by GC proportion and base coverage. Marker area is proportional to scaffold length, and colour indicates taxonomic assignment ( Challis *et al.*, 2020).

**Figure 6.**
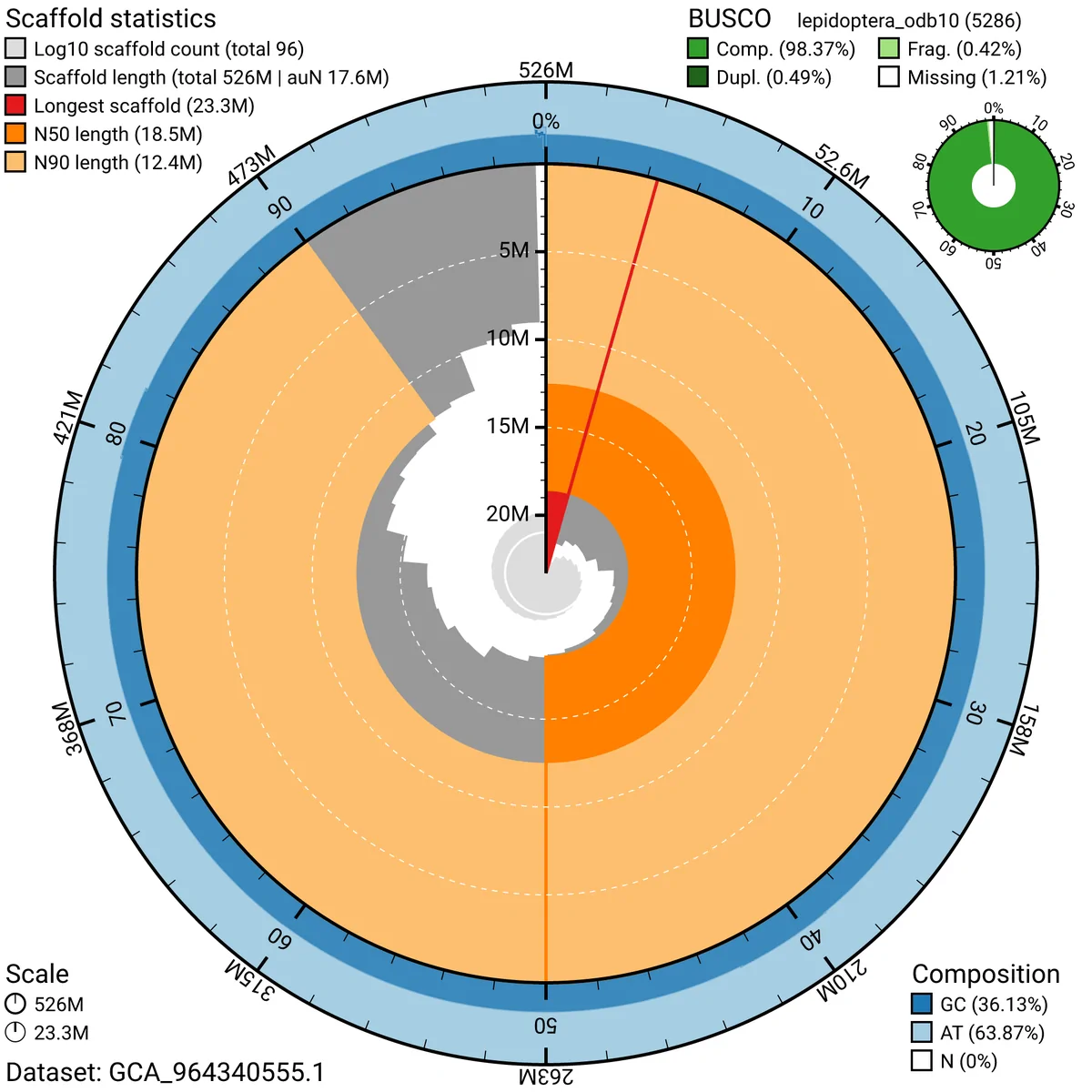
Assembly metrics for ilCabExan1.hap1.1, the haplotype 1 assembly. The BlobToolKit snail plot provides an overview of assembly metrics and BUSCO gene completeness. The circumference represents the length of the haplotype 1 assembly, and the main plot is divided into 1,000 bins around the circumference. The outermost blue tracks display the distribution of GC, AT, and N percentages across the bins. Scaffolds are arranged clockwise from longest to shortest and are depicted in dark grey. Scaffold length is shown on a linear radial scale, scaled to the longest scaffold. Red shading indicates the longest scaffold, and the deeper orange and pale orange shading represent the N50 and N90 lengths. The summary statistics include scaffold auN, the area under the scaffold Nx curve. The light grey spiral at the centre shows the cumulative scaffold count on a logarithmic scale. A summary of complete, fragmented, duplicated, and missing BUSCO genes in the lepidoptera_odb10 set is presented at the top right. The assembly can be explored interactively in the BlobToolKit viewer.

**Figure 7.**
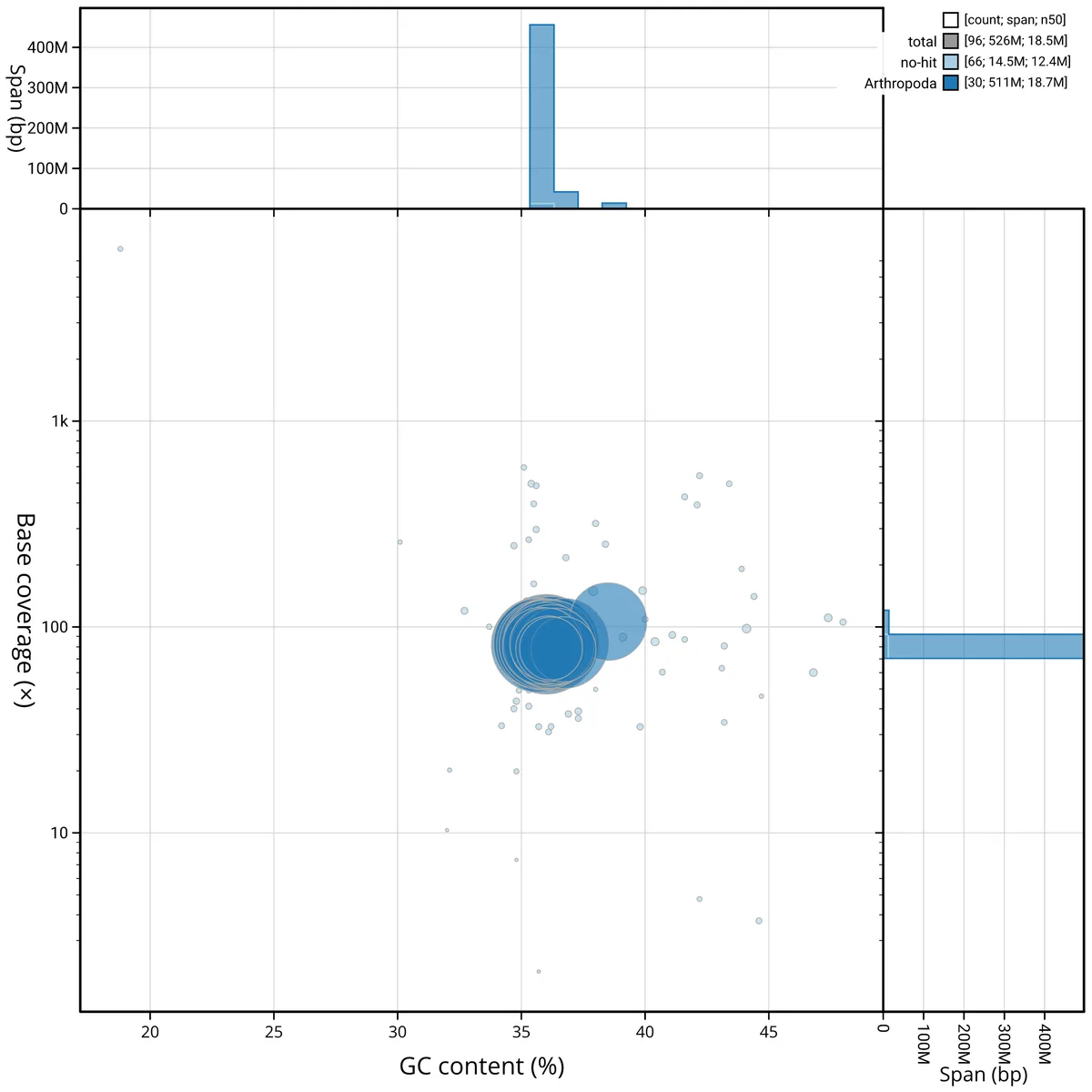
BlobToolKit blob plot for ilCabExan1.hap1.1, the haplotype 1 assembly. The plot shows base coverage (vertical axis) and GC content (horizontal axis). Circles represent scaffolds; marker area is proportional to scaffold length, and colour indicates taxonomic assignment. The histograms along the axes display the total length of sequences distributed across different levels of coverage and GC content. The assembly can be explored interactively in the BlobToolKit viewer.

Table 4 lists the assembly metric benchmarks adapted from Rhie *et al.*. (2021) and the Earth BioGenome Project (EBP) Report on Assembly Standards. The three-part EBP metric, calculated for the haplotype 1 assembly, is **7.C.Q65**.

**Table 4.** Earth BioGenome Project summary metrics for the *C. exanthemata* assembly

**Measure**	**Value**	***Benchmark***
EBP summary (haplotype 1)	7.C.Q65	*6.C.Q40*
Contig N50 length	10.27 Mb	*> 1 Mb*
Scaffold N50 length	18.52 Mb	*= chromosome N50*
Consensus quality (QV)	Haplotype 1: 65.5; haplotype 2: 67.6; combined: 66.4	*≥ 40*
*k*-mer completeness	Haplotype 1: 75.57%; Haplotype 2: 75.52%; combined: 99.70%	*> 90%*
BUSCO	Haplotype 1: C:98.4% [S:97.9%, D:0.5%], F:0.4%, M:1.2%, n:5,286; Haplotype 2: C:98.4% [S:97.9%, D:0.5%], F:0.5%, M:1.2%, n:5,286	*S > 90%; D < 5%*
Percentage of assembly assigned to chromosomes	99.60%	*≥ 90%*

**Notes:** The EBP summary uses log10(Contig N50); chromosome-level (C) or log10(Scaffold N50); Q (Merqury QV). BUSCO v5.5.0 using the lepidoptera_odb10 lineage dataset: C=complete; S=single-copy; D=duplicated; F=fragmented; M=missing; n=orthologues.

### Data availability

European Nucleotide Archive: Cabera exanthemata (common wave). Accession number PRJEB82149; https://identifiers.org/ena.embl/PRJEB82149. The genome sequence is released openly for reuse. The *C. exanthemata* genome sequencing initiative is part of the Darwin Tree of Life Project ( PRJEB40665), the Sanger Institute Tree of Life Programme ( PRJEB43745) and Project Psyche ( PRJEB71705). All raw sequence data and the haplotype assemblies have been deposited in INSDC databases. The genome will be annotated using available RNA-Seq data and presented through the Ensembl pipeline at the European Bioinformatics Institute. Raw data and assembly accession identifiers are reported in Table 1 and Table 2.

Production code used in genome assembly at the WSI Tree of Life is available at https://github.com/sanger-tol. Table 5 lists software versions used in this study.

**Table 5.** Software versions and sources

**Software**	**Version**	**Source**
BLAST	2.14.0	https://ftp.ncbi.nlm.nih.gov/blast/executables/blast+/
BlobToolKit	4.3.9	https://github.com/genomehubs/blobtoolkit
BlobTk (hosted BlobDir)	0.5.1	https://github.com/genomehubs/blobtk
BlobTk (snail and blob plot renderer)	0.8.3	https://github.com/genomehubs/blobtk
BUSCO	5.5.0; 6.1.0	https://gitlab.com/ezlab/busco
busco-alg-painter	0.1.0	https://github.com/Karenvn/busco-alg-painter
bwa-mem2	2.2.1	https://github.com/bwa-mem2/bwa-mem2
DIAMOND	2.1.8	https://github.com/bbuchfink/diamond
fasta_windows	0.2.4	https://github.com/tolkit/fasta_windows
FastK	1.2	https://github.com/thegenemyers/FASTK
GenomeScope	2.1.0	https://github.com/tbenavi1/genomescope2.0
Gfastats	1.3.6	https://github.com/vgl-hub/gfastats
Hifiasm	0.19.8-r603	https://github.com/chhylp123/hifiasm
HiGlass	1.13.4	https://github.com/higlass/higlass
MerquryFK	1.1.0-c1	https://github.com/thegenemyers/MERQURY.FK
Minimap2	2.24-r1122	https://github.com/lh3/minimap2
MitoHiFi	3.2.2	https://github.com/marcelauliano/MitoHiFi
MultiQC	1.14; 1.17 and 1.18	https://github.com/MultiQC/MultiQC
Nextflow	23.10.0	https://github.com/nextflow-io/nextflow
PretextSnapshot	0.0.6	https://github.com/sanger-tol/PretextSnapshot
PretextView	1.0.3	https://github.com/sanger-tol/PretextView
samtools	1.19.2	https://github.com/samtools/samtools
sanger-tol/ascc	0.1.0	https://github.com/sanger-tol/ascc
sanger-tol/blobtoolkit	0.6.0	https://github.com/sanger-tol/blobtoolkit
sanger-tol/curationpretext	1.4.2	https://github.com/sanger-tol/curationpretext
Seqtk	1.4-r122	https://github.com/lh3/seqtk
Singularity	3.9.0	https://github.com/sylabs/singularity
TreeVal	1.1.1	https://github.com/sanger-tol/treeval
YaHS	1.2a.2	https://github.com/c-zhou/yahs

### Author information

Contributors are listed at the following links:

- Members of the Wellcome Sanger Institute Tree of Life Management, Samples and Laboratory team
- Members of Wellcome Sanger Institute Scientific Operations – Sequencing Operations
- Members of the Wellcome Sanger Institute Tree of Life Core Informatics team
- Members of the Tree of Life Core Informatics collective
- Members of the Darwin Tree of Life Consortium

### Wellcome Sanger Institute – Legal and Governance

The materials that have contributed to this genome note have been supplied by a Darwin Tree of Life Partner. The submission of materials by a Darwin Tree of Life Partner is subject to the **‘Darwin Tree of Life Project Sampling Code of Practice’**, which can be found in full on the Darwin Tree of Life website. By agreeing with and signing up to the Sampling Code of Practice, the Darwin Tree of Life Partner agrees they will meet the legal and ethical requirements and standards set out within this document in respect of all samples acquired for, and supplied to, the Darwin Tree of Life Project. Further, the Wellcome Sanger Institute employs a process whereby due diligence is carried out proportionate to the nature of the materials themselves, and the circumstances under which they have been/are to be collected and provided for use. The purpose of this is to address and mitigate any potential legal and/or ethical implications of receipt and use of the materials as part of the research project, and to ensure that in doing so we align with best practice wherever possible. The overarching areas of consideration are:

- Ethical review of provenance and sourcing of the material
- Legality of collection, transfer and use (national and international)

Each transfer of samples is further undertaken according to a Research Collaboration Agreement or Material Transfer Agreement entered into by the Darwin Tree of Life Partner, Genome Research Limited (operating as the Wellcome Sanger Institute), and in some circumstances, other Darwin Tree of Life collaborators.
