# Supplementary material for: The genome sequence of the Common Wave, Cabera exanthemata (Scopoli, 1763) (Lepidoptera: Geometridae)
Source: Wellcome Open Res. 2026 Sep 24;11:736. [Version 1] doi: 10.12688/wellcomeopenres.27917.1 (PMC13694934; doi:10.12688/wellcomeopenres.27917.1)
Supplement: Peer review history

# Reviewer report: Reviewer response for version 1

**DOI:** 10.21956/wellcomeopenres.30717.r175395

**Published:** 2026-11-04

**Authors:** Mar Repulles

**Affiliations:** 1 Zoology, University of South Bohemia in Ceske Budejovice Faculty of Science (Ringgold ID: 204738), Ceske Budejovice, South Bohemian Region, Czech Republic

The manuscript presents a well-described genome assembly of a male *Cabera exanthemata* (Scopoli, 1763), following the established format and methodological standards of the Tree of Life Genome Notes. The assembly comprises two haplotype assemblies, with 99.6% of haplotype 1 assigned to 31 chromosomal pseudomolecules, including the Z chromosome, while haplotype 2 was assembled to the scaffold level. A mitochondrial genome assembly is also provided. The work was conducted as part of the Darwin Tree of Life project.

 The species background is clearly presented, and the manuscript provides the expected genomic information and quality metrics. I have only a few minor suggestions regarding the background section:

- Species names: I suggest including the scientific names of the two similar species mentioned: the Common White Wave (Cabera pusaria) and the Grass Wave (Perconia strigillaria). For consistency with the capitalisation of other species' common names throughout the manuscript, "grass wave" could be changed to "Grass Wave".
- Host plants: The authors could consider mentioning additional reported larval host plants, although these are rare according to the sources: *Betula *spp. and *Alnus* spp. (Jonko, n.d.).
- Minor typographical error: In the Assembly quality metrics section, "Rhie et al.. (2021)" should be corrected to "Rhie et al., (2021)".

Regarding the genomic data, although genome assembly methodology is not my primary area of expertise, I have experience working with genomic datasets and downstream analyses. From this perspective, the assembly and quality assessment procedures appear appropriate, are clearly documented, and follow established workflows used in other Tree of Life

Are the datasets clearly presented in a usable and accessible format, and the assembly and annotation available in an appropriate subject-specific repository?

Yes

Are sufficient details of the sequencing and extraction, software used, and materials provided to allow replication by others?

Yes

Are the rationale for sequencing the genome and the species significance clearly described?

Yes

Are the protocols appropriate and is the work technically sound?

Yes

Reviewer Expertise:

Evolutionary genomics of Lepidoptera.

I confirm that I have read this submission and believe that I have an appropriate level of expertise to confirm that it is of an acceptable scientific standard.

## References

[1] Jonko, C. (n.d.). Lepidoptera Mundi. https://lepidoptera.eu/.

# Reviewer report: Reviewer response for version 1

**DOI:** 10.21956/wellcomeopenres.30717.r175396

**Published:** 2026-10-23

**Authors:** Jullien Flynn

**Affiliations:** 1 université laval, Quebec City, Canada

This article reports the new genome assembly of the Common Wave moth, assembled from PacBio long read sequencing and Hi-C scaffolding. The genome was assembled at the pseudo-chromosomal level and has a haploid size of approximately 525 Mb. The authors report standard metrics of genome assembly completeness and quality. The details of the methods and the accessibility of the data are acceptable.

Are the datasets clearly presented in a usable and accessible format, and the assembly and annotation available in an appropriate subject-specific repository?

Yes

Are sufficient details of the sequencing and extraction, software used, and materials provided to allow replication by others?

Yes

Are the rationale for sequencing the genome and the species significance clearly described?

Yes

Are the protocols appropriate and is the work technically sound?

Yes

Reviewer Expertise:

genomics, genome assemblies, repetitive DNA, Drosophila

I confirm that I have read this submission and believe that I have an appropriate level of expertise to confirm that it is of an acceptable scientific standard.

# Reviewer report: Reviewer response for version 1

**Published:** 2026-09-24

**Authors:** Automated Benchmarking

**Affiliations:** 1 Wellcome Sanger Institute, Hinxton, UK

As this article is a Genome Note, Automated Benchmarking has been used to determine whether the reported assembly passes four defined checks established by the Earth BioGenome Project: the raw and assembled data are publicly available, the Earth BioGenome Project three-part metric is met, the k-mer completeness exceeds 90%, and the BUSCO score passes the stated threshold. For Cabera exanthemata (ilCabExan1), all four checks passed. Data available: pass. ENA study PRJEB82149, raw-read accessions ERR13946452 and ERR13947471, and assembly accessions GCA_964340555.1 and GCA_964340675.1 are public. Earth BioGenome Project three-part metric: pass. Haplotype 1 is reported as 7.C.Q65, meeting the 6.C.Q40 reference standard. k-mer completeness: pass. The combined haplotype assemblies have 99.7% k-mer completeness, exceeding the Earth BioGenome Project target of 90%. BUSCO: pass. Haplotype 1, which is proposed as the reference assembly for this species, has 98.4% complete BUSCOs (97.9% single-copy and 0.5% duplicated). BUSCO software version: 5.5.0; lineage dataset: lepidoptera_odb10. It meets the target of greater than 90% complete and single-copy BUSCOs. This decision uses values reported in the Genome Note and public accession records.

Reviewer Expertise:

NA

I confirm that I have read this submission and believe that I have an appropriate level of expertise to confirm that it is of an acceptable scientific standard.
